# Assessment of the Curative Anti-Glycation Properties of a Novel Injectable Formulation Combining Dual-Weight Hyaluronic Acid (Low- and Mid/High-Molecular Weight) with Trehalose on Human Skin Ex Vivo

**DOI:** 10.3390/ijms26104747

**Published:** 2025-05-15

**Authors:** Robert Chmielewski, Agata Lebiedowska, Wioletta Barańska-Rybak

**Affiliations:** 1 Prime Clinic, Topiel 12, 00-342 Warsaw, Poland; cmvitamed@gmail.com; 2 Positive Pro-Aging Foundation, Topiel 12, 00-342 Warsaw, Poland; 3 URGO Aesthetics Department, URGO Sp. z o.o., Aleje Jerozolimskie 142 B, 02-305 Warsaw, Poland; 4 Department of Basic Biomedical Science, Faculty of Pharmaceutical Sciences in Sosnowiec, Medical University of Silesia, Katowice, Poland, Jednosci 8B, 41-208 Sosnowiec, Poland; 5 Department of Dermatology, Venereology, and Allergology, Faculty of Medicine, Medical University of Gdańsk, Smoluchowskiego 17, 80-214 Gdansk, Poland; wiolabar@gumed.edu.pl

**Keywords:** glycation, hyaluronic acid, trehalose, skin aging, dermal matrix, explants

## Abstract

Glycation influences skin aging through non-enzymatic reactions between reducing sugars and proteins, forming advanced glycation end-products (AGEs) that accelerate skin deterioration. This study evaluates the curative anti-glycation effects of an injectable formulation combining dual-molecular-weight hyaluronic acid (low and mid/high) with trehalose in methylglyoxal-induced glycation in human skin explants. Thirty-six human skin explants were allocated across five experimental groups in a 12-day study. Glycation was induced using methylglyoxal (500 μM) on days 1 and 4, followed by curative product administration on day 5. CML (Nε-(carboxymethyl)lysine) immunohistochemistry was performed to assess glycation levels in the reticular dermis at days 6, 8, and 12, with quantitative analysis conducted through standardized image analysis. The formulation significantly reduced CML formation by 60% on day 6 compared to untreated controls (*p* < 0.001). Under methylglyoxal-induced glycation stress the product showed sustained curative effects, with CML reductions of 69% on day 6 (*p* = 0.008), 68% on day 8 (*p* = 0.012), and 61% on day 12 (*p* = 0.033) compared to methylglyoxal treatment alone. Cell viability remained unaffected throughout the study period across all experimental conditions. The tested injectable formulation exhibits significant and sustained curative anti-glycation properties in human skin explants for 12 days, effectively counteracting methylglyoxal-induced glycation damage without affecting cell viability. These findings advance anti-aging skin interventions, offering a novel approach to address glycation-induced skin damage with potential applications in clinical dermatology and aesthetic medicine.

## 1. Introduction

**Figure 1:**
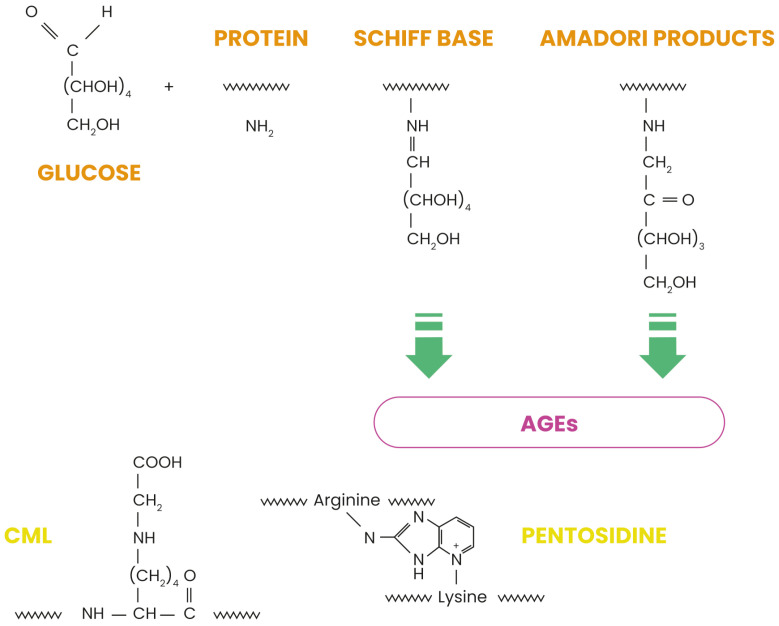
Maillard reaction pathway leading to the formation of advanced glycation end products.

Skin aging is a complex biological process influenced by internal and external factors, with glycation as a critical molecular mechanism contributing to dermal degradation. This non-enzymatic reaction between reducing sugars and proteins forms advanced glycation end-products (AGEs), which accumulate in tissues over time and accelerate structural and functional deterioration. Among these AGEs, Nε-(carboxymethyl)lysine (CML) serves as a biomarker of glycative stress, which is implicated in numerous pathophysiological changes associated with skin aging [1,2]. Unlike enzymatic glycosylation, glycation occurs spontaneously and results in potentially harmful modifications of biomolecules [2,3]. In skin tissues, glycation primarily affects long-lived structural proteins such as collagen and elastin, which constitute the primary components of the dermal extracellular matrix (ECM). The glycation process occurs in several steps, as illustrated in Figure 1. First, reversible Schiff bases form when carbonyl groups of sugars connect with proteins. During this classical Maillard reaction, electrophilic carbonyl groups of glucose or other reactive sugars react with free amino groups, especially basic lysine or arginine residues, forming this non-stable initial compound [4]. These intermediates then undergo Amadori rearrangement, creating more stable products. This rearrangement transforms the unstable Schiff bases into more stable ketoamines (Amadori product), although these products remain reversible reaction intermediates. Further oxidative and non-oxidative reactions generate irreversible AGEs, including CML, pentosidine, and glucosepane [5]. Figure 1 shows the chemical structures of CML and pentosidine, two significant AGEs found in skin tissues. CML forms through the oxidative degradation of Amadori products, while pentosidine creates fluorescent protein–protein crosslinks between arginine and lysine residues. While glycation naturally occurs during aging, factors such as hyperglycemia, oxidative stress, and exposure to glycating agents accelerate it [4,5,6].

At the molecular level, collagen glycation increases intermolecular cross-linking, decreasing solubility, impairing enzymatic degradation, and altering mechanical properties of the dermal matrix. Cross-linked collagen fibers exhibit increased stiffness and reduced elasticity, contributing to the characteristic loss of skin pliability observed in aged skin [7]. At the cellular level, AGEs exert their effects by interacting with specific receptors, most notably the receptor for advanced glycation end-products (RAGE). AGE-RAGE signaling activates multiple downstream pathways, including nuclear factor kappa B (NF-κB), leading to increased production of pro-inflammatory cytokines, matrix metalloproteinases, and reactive oxygen species. This activation has been well-documented in numerous studies using various model systems. While researchers primarily study RAGE signaling pathways in living systems with active cellular responses, evidence suggests that these molecular mechanisms remain partially functional in ex vivo human skin explants during the experimental timeframe, allowing for the meaningful evaluation of anti-glycation interventions in this model [6,8,9,10,11]. However, we acknowledge that the full complexity of RAGE-mediated inflammatory cascades may be attenuated in the explant model compared to in vivo conditions. Despite these methodological considerations, this sustained inflammatory response contributes to the chronic low-grade inflammation characteristic of aged skin and intensifies dermal matrix degradation [10]. The clinical consequences of cutaneous glycation include skin yellowing, deep wrinkle formation, facial contour loss, and decreased skin elasticity. Functionally, skin exhibits a damaged barrier function, reduced hydration, impaired wound healing, and increased susceptibility to stressors [9]. Modern glycation reduction methods have progressed from simple volume procedures to active treatments targeting aging at the molecular level. Injectable anti-glycation treatments offer precise delivery and better penetration into deeper skin layers. Introducing anti-glycation compounds directly into the dermis allows these treatments to bypass the outer skin layer and reach higher concentrations where AGEs accumulate. The formulation evaluated in this study combines different molecular-weight hyaluronic acids with trehalose, with each component selected for its specific biochemical properties and potential anti-glycation effects. Hyaluronic acid (HA) demonstrates several biological activities beyond its volumizing properties, including antioxidant capacity, free radical scavenging, and modulation of inflammatory responses [12]. The HA molecular weight significantly influences its biological properties and tissue penetration, with different molecular weight ranges providing complementary benefits [13,14,15]. Trehalose, a non-reducing disaccharide, represents the second key component of the tested formulation. Unlike reducing sugars that participate in glycation reactions, trehalose lacks a free aldehyde or ketone group, making it unable to initiate the Maillard reaction. It stabilizes protein structure during cellular stress, preventing denaturation and aggregation that might otherwise expose additional glycation-susceptible sites [13]. The rationale for combining different molecular-weight HAs with trehalose stems from their complementary mechanisms against glycation-related damage. Previous studies have demonstrated that high-molecular-weight HA (>1000 kDa) provides excellent water retention and antioxidant properties on the skin surface, while low-molecular-weight HA (≤500 kDa) penetrates deeper into the dermis, stimulating fibroblast activity and enhancing extracellular matrix remodeling [16]. Trehalose protects proteins against various stressors through mechanisms distinct from those of HA, including the prevention of protein unfolding, the inhibition of AGE formation, and the enhancement of cellular autophagy pathways [11,17,18]. Several in vitro studies have demonstrated the synergistic potential of HA and trehalose. Researcher shows that the combination provides superior protection against glycation-induced collagen damage compared to either compound alone [19]. Documentation confirms enhanced skin hydration and elasticity measurements when both compounds work together in dermal applications [19,20]. These findings suggest that the combination may simultaneously address multiple aspects of glycation-related skin damage, providing both protective and reparative effects on the dermal extracellular matrix.

This study aims to evaluate the curative anti-glycation effect of this injectable combination product on human skin explants, focusing particularly on its ability to reduce CML formation following methylglyoxal-induced glycation stress.

## 2. Results

### 2.1. Cell Viability

The curative product (Pc) evaluated in this study combined trehalose with hyaluronic acids of two different molecular weight ranges (low and mid/high) in an injectable formulation. Analysis throughout days 6, 8, and 12, showed that this curative agent did not significantly impact cell viability compared to the control samples.

**Figure 2:**
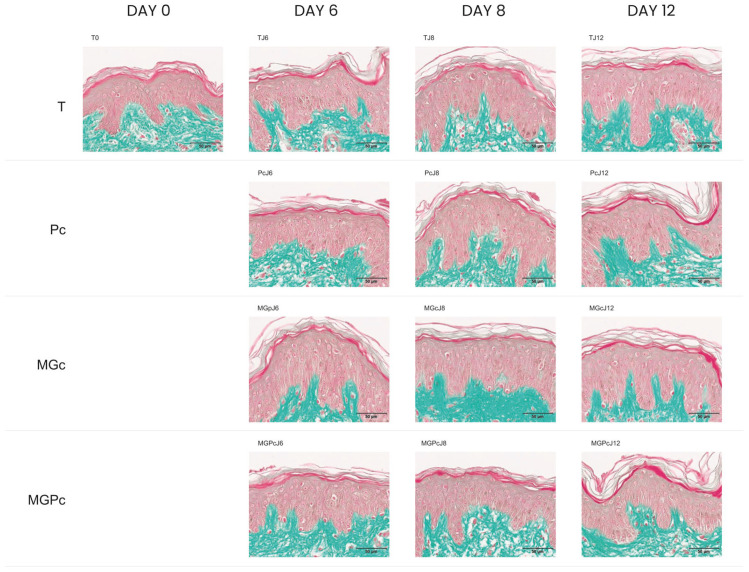
Masson’s trichrome-stained cross-sections of human skin explants. Images represent: baseline sample (T0); untreated control at day 6 (TJ6); Pc-treated sample at day 6 (PcJ6); methylglyoxal-exposed sample at day 6 (MGpJ6); combined Pc and methylglyoxal treatment at day 6 (MGPcJ6); untreated control at day 8 (TJ8); Pc-treated sample at day 8 (PcJ8); methylglyoxal-exposed sample at day 8 (MGcJ8); combined Pc and methylglyoxal treatment at day 8 (MGPcJ8); untreated control at day 12 (TJ12); Pc-treated sample at day 12 (PcJ12); methylglyoxal-exposed sample at day 12 (MGcJ12); and combined Pc and methylglyoxal treatment at day 12 (MGPcJ12). Color identification: epidermal structures—red/pink; dermal collagen fibers—green. Scale 50 µm.

This investigation included several experimental conditions across multiple timepoints: baseline measurements (T0), controls at days 6, 8, and 12 (TJ6, TJ8, and TJ12), samples treated with the curative product (PcJ6, PcJ8, and PcJ12), samples exposed to methylglyoxal as a glycation-inducer (MGpJ6, MGcJ8, and MGcJ12), and samples receiving both treatments (MGPcJ6, MGPcJ8, and MGPcJ12). Cell viability remained unchanged when the curative product was applied under methylglyoxal-induced glycation stress. The data show that the Pc maintained cell viability at levels equivalent to control conditions, neither improving nor diminishing cellular survival under standard or glycation conditions (Figure 2).

### 2.2. Results of CML Immunohistochemical Analysis

**Figure 3:**
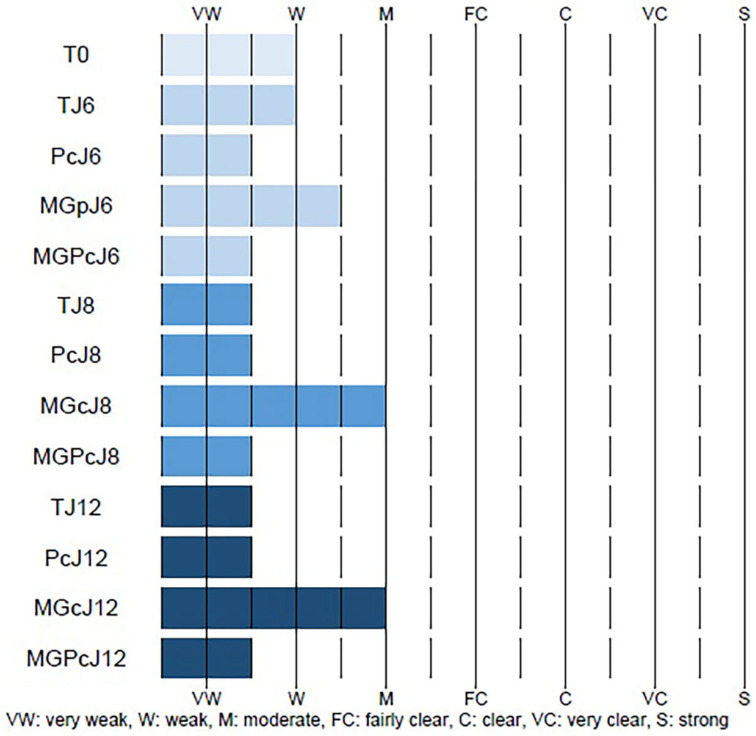
Quantitative assessment of CML (Nε-(carboxymethyl)lysine) immunostaining in the dermis (reticular layer) of human skin explants. The data represent the immunostaining intensity under various experimental conditions: Tm (negative control and no antibody); T0 (baseline); TJ6 (untreated control, day 6); PcJ6 (curative product Pc application, day 6); MGpJ6 (methylglyoxal exposure, day 6); MGPcJ6 (combined Pc and methylglyoxal, day 6); TJ8 (untreated control, day 8); PcJ8 (Pc application, day 8); MGcJ8 (methylglyoxal exposure, day 8); MGPcJ8 (combined Pc and methylglyoxal, day 8); TJ12 (untreated control, day 12); PcJ12 (Pc application, day 12); MGcJ12 (methylglyoxal exposure, day 12); and MGPcJ12 (combined Pc and methylglyoxal, day 12). Staining intensity was scored on a scale ranging from VW (very weak) to S (strong), with intermediate values of W (weak), M (moderate), FC (fairly clear), C (clear), and VC (very clear), where dashed lines indicate boundaries between intensity categories and solid blue bars represent the measured intensity range for each sample (TJ6-MGPcJ12).

At baseline, control samples (T0) showed very weak to weak CML immunostaining intensity in the reticular dermis. Day 6 control samples (TJ6) exhibited similarly low staining intensity. The curative product (Pc) application on day 6 showed minimal changes in CML staining compared to the control. Methylglyoxal (MGp) induction of glycation on day 6 moderately increased CML formation compared to the controls. Importantly, simultaneous application of the curative product with methylglyoxal (MGPc) at day 6 reduced CML staining to levels similar to samples treated with the curative product alone. Day 8 and 12 samples showed a similar pattern. Control samples (TJ8 and TJ12) and curative product-treated samples (PcJ8 and PcJ12) maintained weak staining intensity. Methylglyoxal-treated samples (MGcJ8 and MGcJ12) consistently showed the highest CML formation with moderate intensity. The combination treatment (MGPcJ8 and MGPcJ12) consistently reduced CML immunostaining compared to methylglyoxal treatment alone across all timepoints (Figure 3). These findings suggest that the curative product exhibits consistent anti-glycation activity against glycation-induced conditions in the reticular dermis throughout the experimental timeline, maintaining its protective effect through day 12.

### 2.3. Quantification of CML Immunostaining in Human Skin Explants

**Figure 4:**
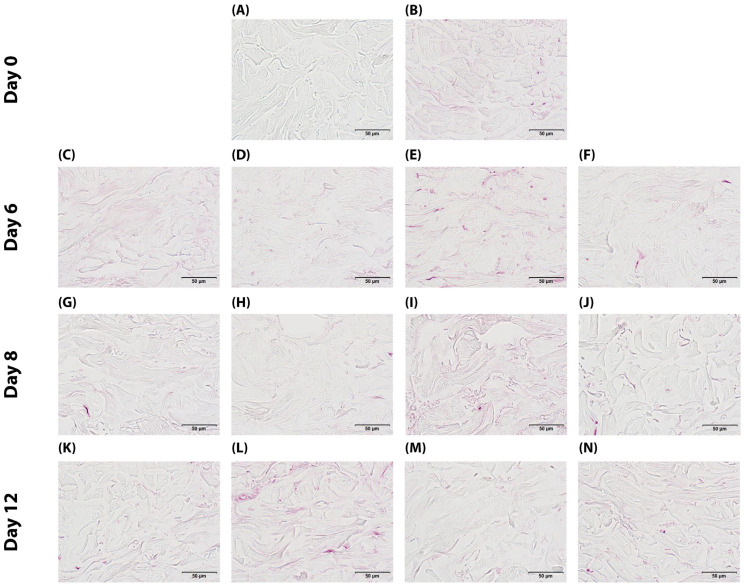
Immunohistochemical detection of CML in human skin explants: (**A**) negative control (no primary antibody) (Tm); (**B**) baseline at day 0 (T0); (**C**) non-treated control at day 6 (TJ6); (**D**) Pc-treated sample at day 6 (PcJ6); (**E**) methylglyoxal-exposed sample at day 6 (MGpJ6); (**F**) dual treatment with Pc and methylglyoxal at day 6 (MGPcJ6); (**G**) non-treated control at day 8 (TJ8); (**H**) Pc-treated sample at day 8 (PcJ8); (**I**) methylglyoxal-exposed sample at day 8 (MGcJ8); (**J**) dual treatment with Pc and methylglyoxal at day 8 (MGPcJ8); (**K**) non-treated control at day 12 (TJ12); (**L**) Pc-treated sample at day 12 (PcJ12); (**M**) methylglyoxal-exposed sample at day 12 (MGcJ12); (**N**) dual treatment with Pc and methylglyoxal at day 12 (MGPcJ12).

The anti-glycation efficacy of the Pc treatment was assessed through the quantitative analysis of CML immunohistochemical staining in the reticular dermis (Figure 4). Baseline samples (T0) exhibited CML immunoreactivity in 4.7% of the reticular dermis surface area. Control samples (TJ6) showed similar levels (5.0% of surface area) after 6 days. Application of the Pc (curative treatment) on day 6 significantly reduced CML formation by 60% (*p* < 0.001) compared to time-matched controls, with the CML-positive area decreasing to 2.0%. Methylglyoxal induction of glycation stress (MGpJ6) significantly increased CML immunoreactivity by 40% (*p* = 0.019) compared to untreated controls, reaching 7.0% of the surface area. Notably, Pc maintained its efficacy under glycation challenge, with the combined treatment (MGPcJ6) showing a significant 69% reduction in CML formation (*p* = 0.008) compared to methylglyoxal treatment alone. At day 8, untreated controls (TJ8) showed CML immunoreactivity in 2.5% of the reticular dermis. Pc treatment (PcJ8) reduced the CML-positive area by 28% (*p* = 0.038) compared to time-matched controls. Methylglyoxal exposure (MGcJ8) dramatically increased CML formation by 268% (*p* = 0.001) compared to day 8 controls, reaching 9.2% of the surface area. The combined treatment (MGPcJ8) demonstrated continued Pc efficacy, with a significant 68% reduction in CML formation (*p* = 0.012) compared to methylglyoxal alone. By day 12, untreated controls (TJ12) exhibited CML immunoreactivity in 3.2% of the reticular dermis surface. Pc treatment (PcJ12) decreased CML formation by 25% (*p* = 0.039) compared to time-matched controls. Sustained methylglyoxal-induced glycation (MGcJ12) increased CML formation by 200% (*p* = 0.001) compared to day 12 controls, with the CML-positive area reaching 9.6%. The combined treatment (MGPcJ12) continued to demonstrate Pc’s anti-glycation efficacy, with a significant 61% reduction in CML formation (*p* = 0.033) compared to methylglyoxal exposure alone. These comprehensive quantitative findings demonstrate that Pc consistently reduces CML formation under both normal and glycation-stressed conditions across all evaluated timepoints. Statistical analysis confirms the significant anti-glycation activity of Pc in the reticular dermis of human skin explants, with the effect maintained throughout the 12-day experimental period (Table 1 and Table S1). A one-way repeated measures ANOVA revealed significant differences between measurements F(12,96) = 19.33; *p* < 0.001; η^2^ = 0.71. Statistically significant differences are when *p* < 0.05 (*) and when *p* < 0.01 (**), as shown in Table S1. The statistical comparison further validates the observed anti-glycation effects of Pc across all experimental timepoints, confirming that Pc treatment significantly reduced CML formation under both normal and glycation-challenged conditions. These statistical findings strengthen the conclusion that Pc exhibits consistent and robust anti-glycation activity in the reticular dermis of human skin explants throughout the experimental period.

## 3. Discussion

The present study provides evidence for the curative anti-glycation effects of an injectable formulation combining hyaluronic acid of dual molecular weight ranges (low and mid/high) with trehalose on methylglyoxal-induced glycation in human skin explants. The results demonstrate a significant and sustained reduction in CML formation, a principal biomarker of glycative stress, indicating potential therapeutic applications for counteracting glycation-related skin aging. Neumann et al. [21] investigated how hyaluronic acid of different molecular weights affects AGE-induced inflammation in macrophages. They discovered that high-molecular-weight HA (>1.2 MDa) inhibited NF-κB activation and pro-inflammatory cytokine expression, while low-molecular-weight HA (<0.5 MDa) lost this protective effect and became pro-inflammatory itself. We evaluated a therapy combining low- and med/high-molecular-weight hyaluronic acid with trehalose, demonstrating a 60–69% reduction in CML formation in the skin. While Neumann showed beneficial effects only with high-molecular-weight HA, our results revealed a synergistic effect when using two molecular weight ranges with trehalose [21]. Our findings reveal a remarkable 60% reduction in CML formation (*p* < 0.001) in the reticular dermis following Pc treatment compared to time-matched controls at day 6, with persistent effects observed throughout the study period. More significantly, when applied following methylglyoxal-induced glycation stress, the formulation reduced CML by 69% at day 6 (*p* = 0.008), 68% at day 8 (*p* = 0.012), and 61% at day 12 (*p* = 0.033) compared to methylglyoxal treatment alone. This sustained efficacy throughout the 12-day experiment suggests that a single application maintains therapeutic anti-glycation activity for an extended period, offering a potentially valuable characteristic for clinical applications. The baseline CML level in control samples (4.7% of reticular dermis surface area) likely represents physiological glycation occurring naturally in human skin. Interestingly, CML levels did not decrease below a certain threshold even with treatment, suggesting the existence of a physiological baseline of AGEs resulting from normal metabolic processes. The test formulation appears to normalize rather than completely eliminate glycation products, bringing CML levels close to this physiological baseline [22]. The complementary properties of the formulation’s components create a multimodal mechanism underpinning the observed anti-glycation effects. The dual-molecular-weight hyaluronic acid approach targets multiple aspects of glycation-related tissue dysfunction. Low-molecular-weight hyaluronic acid likely promotes angiogenesis, enhancing tissue perfusion and temporarily stimulating pro-inflammatory cytokines that “wake up” tissues from the metabolic lethargy characteristic of chronic glycation. This controlled inflammatory response disrupts the persistent inflammatory state associated with glycation and oxidative stress. Concurrently, mid/high-molecular-weight hyaluronic acid functions as an antioxidant buffer while promoting extracellular matrix rehydration. This rehydration restores the functionality of the glycosaminoglycan-rich interstitial space, the primary medium for intercellular communication, nutrient delivery, and metabolic waste removal. Restoration of this environment reactivates dormant fibroblasts inhibited by high-oxidative-stress conditions [14,15,23]. Trehalose, as the third key component, likely initiates autophagy processes in combination with hyaluronic acid, facilitating the removal of metabolic waste products and dysfunctional cellular components accumulated through chronic glycative stress. This “cleaning” of the extracellular space effectively disrupts the self-perpetuating cycle of glycation, oxidative stress, and chronic inflammation [24,25,26,27]. The components work synergistically to restore the regulatory functions of the extracellular matrix by interrupting chronic glycation processes, neutralizing reactive oxygen species, and re-establishing appropriate hydration levels. This environmental restoration enables fibroblasts to resume collagen production and secrete anti-inflammatory cytokines, thus controlling the inflammatory response and initiating physiological repair mechanisms. Notably, the formulation maintained cell viability throughout the study period across all experimental conditions, indicating a favorable safety profile. This observation, combined with its sustained anti-glycation efficacy, suggests potential for clinical application with minimal cellular toxicity concerns [28,29]. In conclusion, our findings demonstrate that the tested injectable formulation exhibits significant curative anti-glycation properties in human skin explants under methylglyoxal-induced glycative stress. The product appears to work through simultaneous restoration of the extracellular matrix environment, disruption of the glycation–oxidative stress–inflammation cycle, and fibroblast functionality reactivation. These effects persist for at least 12 days following a single administration, offering promising therapeutic potential for addressing glycation-related skin aging through a physiologically balanced approach that targets the fundamental mechanisms of glycative tissue damage.

## 4. Materials and Methods

This investigation was performed in accordance with good laboratory practice principles (Decree of 10 August 2004), while adhering to the validated protocols and standard operating procedures established by Eurofins BIO-EC.

### 4.1. Schedule of the Study

**Figure 5:**
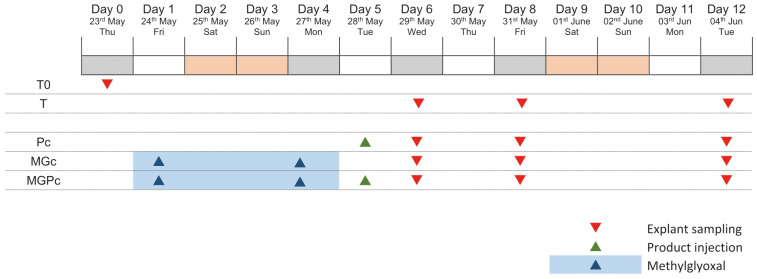
Study design of human skin explant glycation protocol. Red triangles: explant sampling; green triangles: Pc (curative product) injection; blue triangles and shading: methylglyoxal application. T0: baseline; T: untreated control; Pc: curative product group; MGc: methylglyoxal group; MGPc: combined methylglyoxal and curative product group.

The experimental design covers a 12-day study period using human skin explants to evaluate anti-glycation effects. The protocol begins with baseline sample collection (T0) on the initial day. The study includes four distinct experimental groups: an untreated control group (T) maintained throughout the experiment; a Pc-treated group receiving the curative product on day 5; a methylglyoxal-challenged group (MGc) exposed to the glycating agent on days 1 and 4; and a combination group (MGPc) receiving both methylglyoxal treatment on days 1 and 4 followed by Pc administration on day 5. Tissue sampling occurs at three critical timepoints—days 6, 8, and 12—allowing assessment of both immediate and sustained effects across all treatment conditions. This methodical timeline enables comparative evaluation of CML formation under normal conditions, glycation stress, and with potential protective intervention, providing comprehensive assessment of the curative product’s efficacy against glycation-induced changes in human skin explants at different intervals following treatment (Figure 5).

### 4.2. Tested Product

A total of 24 human skin explants with an approximately 11 mm (±1 mm) diameter obtained from abdominal surgery of a 28-year-old female Caucasian donor (identification: P2980-AB28) with Fitzpatrick phototype III classification were used. Following procurement on day 0, the explants were maintained in BIO-EC’s explants medium (BEM) at 37 °C under controlled conditions (humid environment with 5% CO_2_) to ensure tissue viability. This research used surgical tissue residues from cosmetic procedures from a single donor, conducted in full compliance with the Declaration of Helsinki principles and in accordance with article L.1243-4 of the French Public Health Code, which states that ethics committee approval is not mandatory for studies using surgical waste materials.

### 4.3. Explants Sampling

The experimental design incorporated both control conditions and curative intervention assessment groups. Thirty-six human skin explants were systematically distributed across five distinct treatment categories. For control conditions, three explants were allocated to the baseline tissue control group (T0) collected at day 0, while nine explants were designated as non-treated controls (T), with three explants sampled at each of the three evaluation timepoints (days 6, 8, and 12) (Table 2).

For the assessment of curative efficacy, twenty-four explants were distributed across three experimental treatment protocols. The Pc group (nine explants) received the curative product, with three samples evaluated at each timepoint (days 6, 8, and 12). The methylglyoxal-exposed group (MGc) comprised six explants, with three samples collected at each of the later timepoints (days 8 and 12). The combination treatment group (MGPc) included nine explants receiving both the curative product and methylglyoxal challenge, with three samples analyzed at each timepoint (days 6, 8, and 12) (Table 3). This comprehensive allocation strategy enabled evaluation of both normal aging progression and the curative potential of the test product against methylglyoxal-induced glycation in human skin explants across multiple timepoints.

### 4.4. Treatment Protocol

For the curative effect assessment (Pc and MGPc groups), 30 μL of the test formulation (Resteeme X; supplied by URGO Sp. zo.o., Warsaw, Poland) was injected intradermally on day 5 (following completion of the glycation induction phase). Injections were performed using the sponsor-provided syringe after allowing the product to equilibrate to room temperature. The untreated control groups (T) received no interventions beyond standard medium maintenance. The culture medium was partially refreshed (1 mL) on days 1, 4, 8, and 11, with complete renewal (2 mL) occurring on day 5.

### 4.5. Induction of Glycation

To establish glycation conditions in the designated groups (MGc and MGPc), methylglyoxal was added to the BEM culture medium at a final concentration of 500 μM on both day 1 and day 4 of the experiment. On day 5, the entirety of the culture medium for these methylglyoxal-treated groups was replaced with 2 mL of fresh BEM without methylglyoxal. Subsequent medium renewals through day 11 also used methylglyoxal-free medium.

### 4.6. Sample Collection

Explant collecting followed a predetermined schedule across the experimental timeline. On day 0, three explants from the baseline group (T0) were collected and bisected, with one half preserved in buffered formalin for histological analysis and the other half flash-frozen at −80 °C for biochemical evaluation. On day 6, three explants from each relevant group (T, Pc, and MGPc) were collected and processed using identical methodology to the baseline samples. The MGc group was not sampled at this timepoint. On days 8 and 12, three explants from each experimental group (T, Pc, MGc, and MGPc) were collected and processed following the same protocol established for the baseline samples. The treatment and sampling schedule was adjusted as necessary to align with standard working days, in accordance with provisions outlined in the study protocol.

### 4.7. Histological Methods

Following a 24 h fixation period in buffered formalin, tissue samples underwent automated dehydration and paraffin impregnation using a Leica PEARL processor. Sample embedding was performed with a Leica EG 1160 embedding station. Tissue sectioning was conducted using a Leica RM 2125 Minot-type microtome (Wetzlar, Germany) to produce uniform 5-μm thickness sections. These sections were mounted on Superfrost^®^ histological slides (Thermo Scientific, Waltham, MA, USA) for subsequent staining and analysis. Microscopic examination used either a Leica DMLB or an Olympus BX43/BX63 microscope. Digital documentation of histological findings employed an Olympus DP digital camera system with image acquisition and management via Olympus cellSens 4.3 software (Olympus Evident, Rungis, France).

#### 4.7.1. Viability Assessment

Explant viability was monitored in all experimental groups and involved examination of formalin-fixed paraffin-embedded (FFPE) skin sections stained with Masson’s trichrome (Goldner variant). Microscopic examination assessed the cellular integrity and tissue architecture of both epidermal and dermal components to confirm sample viability throughout the experimental period.

#### 4.7.2. CML Detection and Quantification

CML immunohistochemistry covered FFPE skin sections across all experimental groups. The procedure used a monoclonal anti-CML antibody (TransGenic (Chuo-ku, Japan) ref. KH011, clone CMS-10) at 1:50 dilution in PBS-BSA 0.3%, applied overnight at room temperature. The Vectastain kit vector avidin/biotin system amplified signals, with subsequent visualization using VIP chromogen (Vector, ref. PK-7200, Vector Laboratories, Inc., Newark, CA, USA), a peroxidase substrate that produces a violet reaction product upon oxidation.

Immunostaining intensity evaluation combined microscopic assessment with semi-quantitative image analysis using cellSens software (Olympus). Analysis focused specifically on regions of interest (ROI) within the reticular dermis. Each experimental group provided comprehensive assessment of CML formation patterns.

### 4.8. Image Analysis Protocol

**Figure 6:**
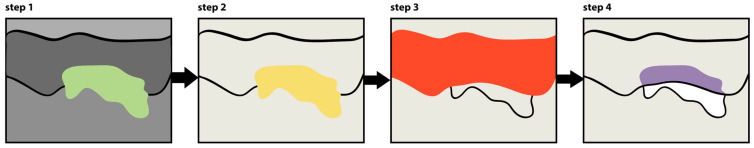
Schematic representation of the image analysis protocol for CML (Nε-(carboxymethyl)lysine) immunostaining quantification in human skin explants. The color scheme represents: green (step 1)—CML-positive areas; yellow (step 2)—mask of detected immunopositive areas; red (step 3)—region of interest (epidermis); purple (step 4)—immunopositive areas within the epidermis.

Quantitative assessment of immunohistochemical staining covered all images from each experimental group using cellSens software (Olympus). The analysis followed a standardized six-step procedure (Figure 6). Digital capture of immunostained tissue sections started the analytical work, with CML-positive areas appearing as green-colored signals in the microscopic field (Step 1). The second analytical step detected staining through threshold-based pixel selection, generating a yellow mask corresponding to all immunopositive areas (Step 2). Step three involved manual delineation of the region of interest (ROI)—in this case, the epidermis—creating a defined measurement area highlighted by a red mask (Step 3). In step four, the software performed an overlay operation between the ROI boundary (red mask) and the detected immunostaining (yellow mask), producing a purple visualization representing the intersection of these two parameters—specifically identifying CML-positive areas within the epidermis (Step 4). Step five calculated surface area measurements, determining both the total ROI area (red mask) and the immunopositive area within that region (purple mask), enabling computation of the percentage of epidermis displaying CML immunoreactivity (Step 5). Finally, step six exported the quantitative results to Excel for statistical processing and comparative analysis between experimental groups (Step 6). This methodology provided objective quantification of CML formation patterns across the various experimental conditions, allowing for statistical comparison between methylglyoxal-exposed samples and either untreated controls or product-treated specimens.

### 4.9. Statistical Analysis

Comparative analysis between experimental groups used repeated measures analysis of variance (ANOVA) followed by post hoc Bonferroni testing to evaluate specific differences between individual measurements. Statistical significance followed conventional threshold criteria; differences between experimental groups were considered statistically significant when *p* < 0.05 (*), representing a 95% confidence level in the observed difference, and highly significant when *p* < 0.01 (**), corresponding to a 99% confidence level. Results that did not meet these threshold criteria were classified as non-significant (NS). This statistical approach provided a rigorous framework for identifying meaningful differences between treatment conditions while controlling for multiple comparisons. IBM SPSS Statistics 23 (2015) processed all statistical data.

## 5. Conclusions

Based on the experimental results, the tested product (P) demonstrated anti-glycation properties when administered as a curative treatment. Cell viability remained unaffected across all timepoints (days 6, 8, and 12) regardless of whether being compared to untreated controls (Ts) or methylglyoxal-exposed samples (MGcs). Regarding CML formation in the reticular dermis, the product exhibited substantial efficacy when administered on day 5 following methylglyoxal-induced glycation. When compared to untreated controls, the product reduced CML levels by 60% on day 6, with persistent reductions of 29% and 25% on days 8 and 12, respectively. Most notably, when compared to methylglyoxal-treated samples, the product demonstrated powerful and sustained anti-glycation activity, with reductions in CML formation of 69% on day 6, 69% on day 8, and 61% on day 12. This indicates that the product maintains its curative anti-glycation efficacy for 12 days following administration, effectively counteracting methylglyoxal-induced glycation damage in human skin explants.

These findings suggest promising clinical potential for this hyaluronic acid–trehalose combination as an injectable anti-glycation intervention for addressing age-related skin changes. The sustained efficacy observed in our ex vivo model indicates potential long-lasting benefits in clinical applications, which could translate to extended intervals between treatments. Future research should focus on evaluating the long-term efficacy and safety in various patient populations. Additionally, comparative studies with existing anti-aging treatments would help to establish the relative efficacy of this approach in the broader context of dermatological interventions for skin aging.

## 6. Patents

“Injectable composition including hyaluronic acid and use of the said composition” US 20210315804A1 [30].

## Figures and Tables

**Table 1:** Quantitative analysis of CML immunoreactivity in human skin explant reticular dermis across experimental timepoints and treatments. Data presented as percentage of CML-positive surface area (mean ± SD) under various conditions: T0 (baseline); TJ6, TJ8, and TJ12 (untreated controls at days 6, 8, and 12); PcJ6, PcJ8, and PcJ12 (Pc treatment at days 6, 8, and 12); MGpJ6, MGcJ8, and MGcJ12 (methylglyoxal exposure at days 6, 8, and 12); MGPcJ6, MGPcJ8, and MGPcJ12 (combined Pc and methylglyoxal treatment at days 6, 8, and 12). Values represent mean and standard deviation from three explants per condition.

	T0	TJ6	PcJ6	MGpJ6	MGPcJ6	TJ8	PcJ8	MGcJ8	MGPcJ8	TJ12	PcJ12	MGcJ12	MGPcJ12
Mean	4.7	5.0	2.0	7.0	2.2	2.5	1.8	9.2	3.2	3.2	2.4	9.6	3.7
SD	1.7	0.9	0.9	2.4	1.4	1.1	2.0	3.6	0.6	0.6	1.3	3.2	1.0

**Table 2:** Distribution of human skin explants in control conditions, detailing batch designation, experimental treatment, number of explants per sampling point, and evaluation timepoints.

Control (Untreated) Condition			
Batch	Conditions	Nb of Explants	Sampling Day
T0 *	Tissue control	3	0
T **	Non-treated control	3, 3, 3	6, 8, 12

* T0: Baseline tissue control samples collected on day 0 (start of the experiment). ** T: Non-treated control samples maintained without intervention and collected at three evaluation timepoints.

**Table 3:** Distribution of human skin explants for curative effect evaluation, specifying batch designation, treatment intervention, number of explants per sampling point, and assessment timepoints.

Curative Effect			
Batch	Conditions	Nb of Explants	Sampling Time
Pc	Injection of product	3, 3, 3	Day 6, day 8, day 12
MGc	Methylglyoxal	3, 3	Day 8, day 12
MGPc	Injectable product + Methylglyoxal	3, 3, 3	Day 6, day 8, day 12

## Data Availability

Data is contained within the article and Supplementary Materials.

## Supplementary Materials

The following supporting information can be downloaded at: https://www.mdpi.com/article/10.3390/ijms26104747/s1.

## Abbreviations

The following abbreviations are used in this manuscript:

MW	Molecular Weight
AGEs	Advanced Glycation End-products
CML	Nε-(carboxymethyl)lysine
ECM	extracellular matrix
RAGE	receptor for advanced glycation end-products
NF-κB	Nuclear factor kappa-light-chain-enhancer of activated B cells
HA	Hyaluronic acid
Pc	curative product
TJ6	control samples on day 6
T0	initial timepoint
TJ8	control samples on day 8
TJ12	control samples on day 12
PcJ6	curative product treatment on day 6
PcJ8	curative product treatment on day 8
PcJ12	curative product treatment on day 12
MGpJ6	glycation-inducing stressor methylglyoxal on day 6
MGcJ8	glycation-inducing stressor methylglyoxal on day 8
MGcJ12	glycation-inducing stressor methylglyoxal on day 12
MGPcJ6	combined treatment with curative product and glycation-inducing methylglyoxal on day 6
MGPcJ8	combined treatment with curative product and glycation-inducing methylglyoxal on day 8
MGPcJ12	combined treatment with curative product and glycation-inducing methylglyoxal on day 12
FFPE	formalin-fixed paraffin-embedded
ROI	region of interest
