# Supplementary material for: Assessment of the Curative Anti-Glycation Properties of a Novel Injectable Formulation Combining Dual-Weight Hyaluronic Acid (Low- and Mid/High-Molecular Weight) with Trehalose on Human Skin Ex Vivo
Source: Int J Mol Sci. 2025 May 15;26(10):4747. doi: 10.3390/ijms26104747 (PMC12111894; doi:10.3390/ijms26104747)
Supplement: Supplementary file 1 [file ijms-26-04747-s001.zip › ijms-3591964-supplementary.pdf]

Supplementary Materials

Table S1. Statistical analysis of CML immunostaining comparisons between experimental conditions in human skin explants. The table presents p-values from multiple comparisons across different time points and treatments: T0 (baseline); TJ6, TJ8, TJ12 (untreated controls at days 6, 8, and 12); PcJ6, PcJ8, PcJ12 (Pc treatment at days 6, 8, and 12); MGpJ6, MGcJ8, MGcJ12 (methylglyoxal exposure at days 6, 8, and 12); MGPcJ6, MGPcJ8, MGPcJ12 (combined Pc and methylglyoxal treatment at days 6, 8, and 12). Statistically significant differences when p<0.05 (*), when p<0.01 (**). Values were derived from analysis of three explants per condition.

	**T0**	**TJ6**	**PcJ6**	**MGpJ6**	**MGPcJ6**	**TJ8**	**PcJ8**	**MGcJ8**	**MGPcJ8**	**TJ12**	**PcJ12**	**MGcJ12**	**MGPcJ12**
**T0**		1.000	0.312	1.000	0.794	1.000	0.510	0.258	1.000	1.000	0.356	0.603	1.000
**TJ6**	1.000		0.000**	1.000	0.006**	0.002**	0.038	1.000	0.009**	0.021*	0.002**	0.676	0.134
**PcJ6**	0.312	0.000**		0.019*	1.000	1.000	1.000	0.058	1.000	0.561	1.000	0.028*	0.005**
**MGpJ6**	1.000	1.000	0.019*		0.008**	0.007**	0.001**	1.000	0.012*	0.057	0.001**	1.000	0.073
**MGPcJ6**	0.794	0.006**	1.000	0.008**		0.794	0.006**	1.000	0.008**	0.794	0.006**	1.000	0.008**
**TJ8**	1.000	0.002**	1.000	0.007**	0.794		1.000	0.107	1.000	0.900	1.000	0.039*	1.000
**PcJ8**	0.510	0.038*	1.000	0.001**	0.006**	1.000		0.087	1.000	1.000	1.000	0.075	0.099
**MGcJ8**	0.258	1.000	0.058	1.000	1.000	0.107	0.087		0.129	0.100	0.080	1.000	0.269
**MGPcJ8**	1.000	0.009**	1.000	0.012*	0.008**	1.000	1.000	0.129		1.000	1.000	0.033*	1.000
**TJ12**	1.000	0.021*	0.561	0.057	0.794	0.900	1.000	0.100	1.000		1.000	0.039*	1.000
**PcJ12**	0.356	0.002**	1.000	0.001**	0.006**	1.000	1.000	0.080	1.000	1.000		0.067	0.042*
**MGcJ12**	0.603	0.676	0.028*	1.000	1.000	0.039*	0.075	1.000	0.033*	0.039*	0.067		0.195
**MGPcJ12**	1.000	0.134	0.005**	0.073	0.008**	1.000	0.099	0.269	1.000	1.000	0.042*	0.195	
